# Unusual site of pseudomyxoma peritonei recurrence after cytoreductive surgery and hyperthermic intraperitoneal chemotherapy: a case report of intraluminal disease manifestation in the small bowel

**DOI:** 10.1186/s12957-022-02613-2

**Published:** 2022-05-10

**Authors:** Paulien Rauwerdink, Lodewijk A. A. Brosens, Karin K. van Diepen, Okan N. Ghedri, Onno Kranenburg, Djamila Boerma, Arjen J. Witkamp, Helma M. U. van Grevenstein

**Affiliations:** 1 Division of Imaging and Cancer, University Medical Centre Utrecht, Room G.04.2.28, PO Box 85500, 3508 GA Utrecht, The Netherlands; 2 Department of Surgery, St. Antonius Hospital, 3435 CM Nieuwegein, The Netherlands; 3 Department of Pathology, University Medical Centre Utrecht, 3508 GA Utrecht, The Netherlands

**Keywords:** Pseudomyxoma peritonei, High-grade appendiceal mucinous neoplasm, CRS-HIPEC, Recurrence, Small bowel, *SMAD4*

## Abstract

**Background:**

Pseudomyxoma peritonei (PMP) is an uncommon clinical condition characterized by the presence of mucinous ascites, mainly induced by perforated appendiceal mucinous neoplasms (AMN). The peritoneal surface of the small bowel is usually spared from disease manifestation due to peristaltic movements. Mucinous tumours can disseminate as PMP on the entire peritoneum, but are rarely intraluminal. For the first time in literature, we report a case of intraluminal PMP involving the ileum.

**Case presentation:**

A 75-year-old male was treated for perforated AMN and disseminated PMP with cytoreductive surgery and hyperthermic intraperitoneal chemotherapy. During follow-up, the patient developed intraperitoneal recurrence together with intraluminal depositions in the ileum, both disease manifestations with identical *KRAS* and *SMAD4* mutations. Hereafter, the patient was treated with palliative care.

**Conclusion:**

This case illustrates the variation in the biological and clinical behaviour of this rare disease. Clinicians should be aware of unusual tumour distribution patterns of PMP, including the presence of mucinous tumour within the small bowel.

## Background

Pseudomyxoma peritonei (PMP) is a rare disease characterized by the presence of mucinous ascites produced by peritoneal implants [1, 2]. This clinical entity most commonly originates from ruptured mucinous appendiceal neoplasms, causing the dissemination of tumour cells throughout the peritoneal cavity [3]. The gradual accumulation of mucinous ascites leads to variations in clinical presentation, with patients often being asymptomatic until an advanced stage is reached [4].

Patients with both early onset or advanced PMP can be treated with curative intent by cytoreductive surgery and hyperthermic intraperitoneal chemotherapy (CRS-HIPEC) [5, 6]. Cytoreduction aims to resect all macroscopic disease to reduce intra-abdominal tumour burden, while the subsequent HIPEC eradicates microscopic residual tumour cells [7, 8]. This combined treatment modality has led to 10-year overall survival reaching 60% [9–11]. Nevertheless, approximately half of all patients will develop recurrent disease [11, 12].

Interestingly, the intestinal peritoneal surfaces are usually spared from disease due to peristaltic movements. As a result of intraperitoneal fluid circulations and gravity, abdominal structures such as the subphrenic and hepatic regions, and the pelvis are at increased risk of being exposed to circulating tumour cells, also known as the redistribution phenomenon [13, 14]. These areas can be technically difficult to ensure complete debulking [10, 15, 16]. Nevertheless, the gradual yet progressive accumulation of mucinous ascites will eventually limit intestinal movements. In end-stage disease, adherence of tumour cells to small bowel surfaces is inevitable and can cause bowel obstruction [2].

To the best knowledge of the authors, no cases of tumour presence within the mucosal wall of the small bowel have been reported. The following case represents an extremely rare location of intraluminal PMP originating from a mucinous appendiceal neoplasm. In this paper, we share our experience with this unusual dissemination pattern.

## Case presentation

A 75-year-old male was referred for unexplained symptoms of nocturia, polyuria, and unintentional weight loss in October 2018. Relevant medical history reported abdominal trauma with hepatic and splenic lacerations, requiring two laparotomies and splenectomy. Upon outpatient assessment, the patient reported occasional pain in the lower abdomen before flatulency. Abdominal examination revealed a small, palpable mass superficially located in the laparotomy scar site and a second, soft, non-tender mass in the left lower quadrant of the abdomen.

**Fig. 1:**
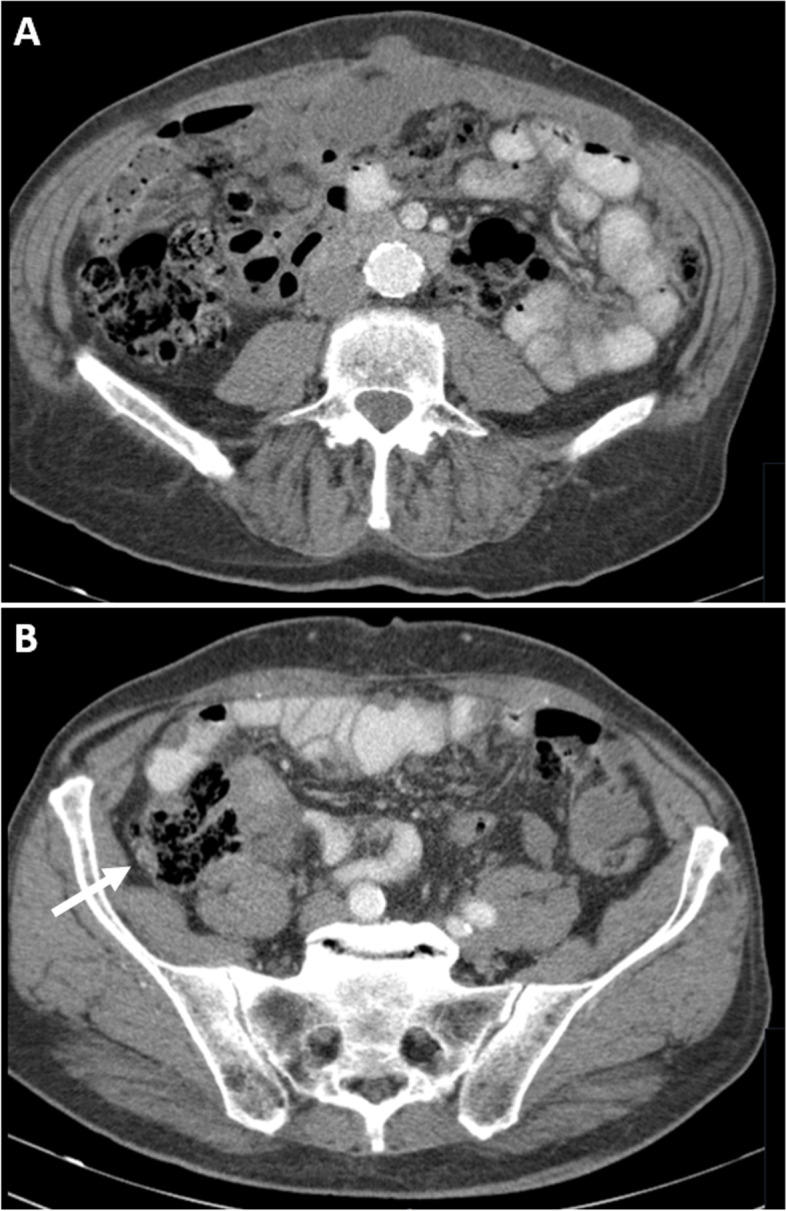
Abdominal CT scans of initial presentation. Cross-sectional images showing mucinous ascites and tumour infiltration of the mesentery and abdominal wall (**A**), and an indistinct cecum mass, indicated by the white arrow (**B**)

Upon diagnostic workup, blood test results showed a c-reactive protein (CRP) level of 26.4 mg/L and elevated gamma-glutamyl transferase (GGT) of 95 U/L. No other biochemical deviations were observed in complete blood counts, renal-, or liver function tests. A thoracoabdominal computed tomography (CT) scan revealed ascites, multiple intraperitoneal tumour depositions and tumour involvement of the small bowel mesentery (Fig. 1a). Tumour obstruction of the distal ureters caused bilateral hydronephrosis. No signs of distant metastases were detected. An indistinct mass in the caecum was presumed to be the primary tumour (Fig. 1b). Subsequent colonoscopy did not identify any (mucosal) abnormalities in the cecum, appendix or elsewhere in the colon.

Ultrasound-guided percutaneous biopsy of an abdominal wall lesion was executed and histopathological examination identified localization of pseudomyxoma peritonei, with immunohistochemical staining being positive for cytokeratin 20 (CK-20) and CDX-2 and negative for cytokeratin 7 (CK-7). Hereafter, the patient was referred to our hospital, a tertiary centre, for further treatment. Tumour markers were assessed (CA 19.9 of 470 U/mL, CEA of 25 μg/L), and PMP originating from an appendiceal mucinous neoplasm was suspected. Eligibility for cytoreductive surgery (CRS) was determined during a multidisciplinary tumour board discussion.

After general anaesthesia, bilateral ureteric stents were placed by the urologist. Upon explorative median laparotomy, bulky tumour was encountered in the abdominal midline. As high-grade disease was suspected, the tumorous tissue was resected and sent for frozen section procedure. The pathological assessment confirmed a low-grade mucinous neoplasm. The extent of peritoneal involvement was assessed by using the Peritoneal Cancer Index (PCI) and scored as 30 [17]. Given the low-grade aspect of frozen section biopsies and the inevitable risk of bowel obstruction due to multiple colonic tumour depositions, it was decided to continue with maximal tumour debulking and subsequent HIPEC. During debulking, a greater omentectomy, peritonectomy of peritoneum overlying the bladder and subtotal colectomy were performed. Mucus located on small bowel serosa and mesentery was removed manually and no serosal injuries occurred. The achieved completeness of cytoreduction (CCR) was defined as CCR2, indicating residual tumor nodules between 0.25 and 2.5 cm [17]. Following cytoreduction, hyperthermic intraperitoneal chemotherapy was applied using the open Coliseum technique with Mitomycin C for 90 min at 42°C, according to the standardized Dutch CRS-HIPEC protocol [18]. After perfusion, an end ileostomy was created and a pelvic drain was placed.

The postoperative course was complicated by a delirium requiring medical treatment (grade 1 complication according to the Clavien-Dindo [CD] classification) and urinary retention, requiring replacement of a long-term catheter (CD grade 2) [19, 20]. The patient was discharged on the ninth postoperative day. Shortly after, the patient was readmitted with clinical symptoms of pyelonephritis, confirmed by positive urinalysis and abdominal CT scan, requiring treatment with oral antibiotics (CD grade 2 complication). Six weeks postoperatively, ureteric stents were removed.

### Histopathological classification

**Fig. 2:**
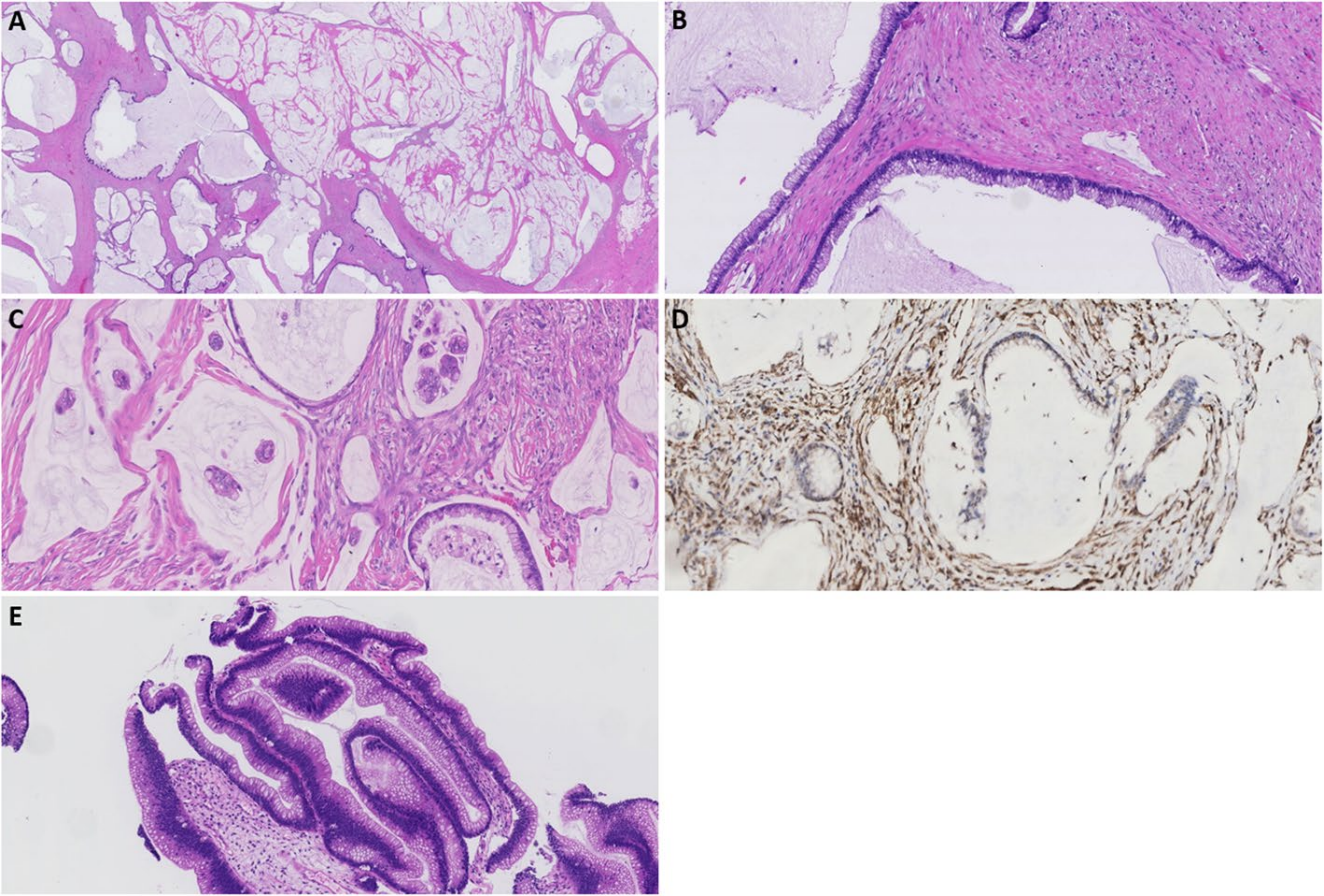
Histopathological images of surgical specimens: **A** Overview of peritoneal disseminated mucinous neoplasm showing by copious mucin pools with scattered strips of mucinous epithelium. **B** Low-grade cytologic atypia with basally located small round nuclei in the peritoneal disseminated mucinous neoplasm. **C** High-grade cytologic grade atypia showing increased nuclear-cytoplasmic ratio in the peritoneal disseminated mucinous neoplasm. **D** SMAD4 immunohistochemistry showing loss of SMAD4 expression in the peritoneal disseminated mucinous neoplasm, consistent with the presence of *SMAD4* mutation. **E** Biopsy of intraluminal recurrence with low cytologic grade atypia

Postoperative histopathological examination demonstrated extensive localization of extracellular mucin in all resected specimens with the presence of mucinous epithelium with mainly mild to moderate cytological atypia (Fig. 2a, b), and focal areas with high-grade cytological atypia and architectural features (Fig. 2c). The appendix was identified after cross-sectioning of the cecum. A high-grade appendicular mucinous neoplasm (HAMN) was encountered according to the PSOGI 2016 consensus classification [3, 6]. In addition, one tubular adenoma with low-grade dysplasia was found in the descending colon and 16 lymph nodes, all without metastases. Based on the clinical and histopathological findings, the final diagnosis was high-grade PMP originating from a HAMN [6].

### Follow-up

In our centre, a thoracoabdominal CT is performed routinely 3, 6, and 12 months after the CRS-HIPEC procedure and every 6 months thereafter, up to 5 years after the initial surgery. The first postoperative CT scan identified multiple peritoneal depositions located cranial-ventral in the hepatic region, with suspicion of surface implants in segment III. Furthermore, extensive peritoneal involvement was seen in the upper left abdominal region. Left-sided pleural effusion was present, without signs of pulmonary metastases. Tumour markers were elevated (CA 19.9 of 160 U/mL, CEA of 10 μg/L). Upon multidisciplinary tumour board discussion, the patient was not considered eligible for re-CRS-HIPEC due to prior comorbidity and progressive disease. Active surveillance and watchful waiting were discussed, to further monitor the patient’s condition.

Seven months after initial surgery, the patient was admitted to the hospital with severe anaemia (haemoglobin of 3.4 mmol/L, MCV of 69fL, ferritin of 7μg/L), without signs of hematemesis or objectified melena. An esophagogastroduodenoscopy excluded gastro-intestinal bleedings. Tumour markers were increased (CA 19.9 of 760 U/mL, CEA of 28 μg/L) and a thoracoabdominal CT scan showed progression of known mucinous depositions in the hepatic and epigastric region.

**Fig. 3:**
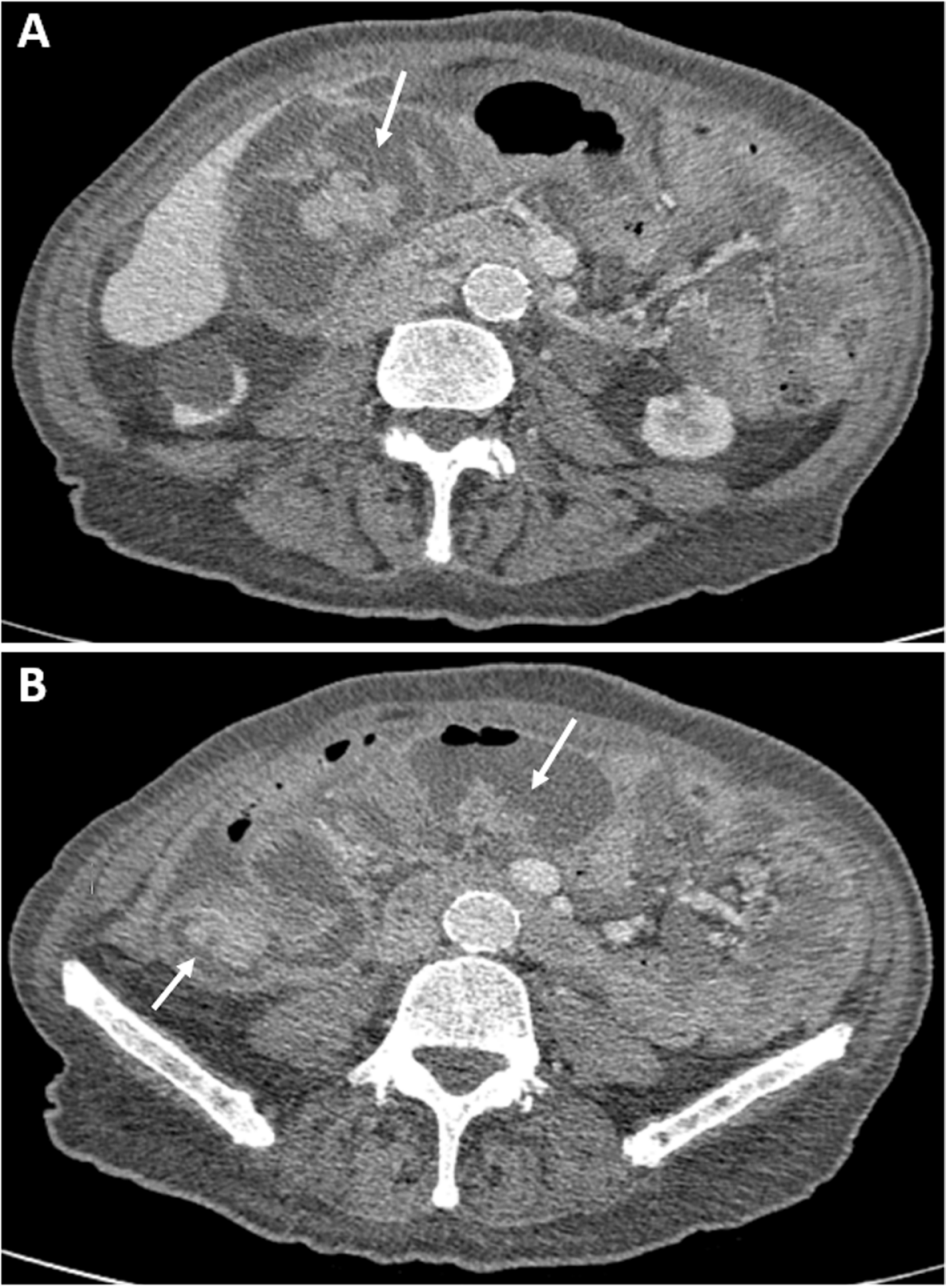
Abdominal CT scan of recurrent disease. Cross-sectional images showing dilation of multiple loops of the small bowel and intraluminal mucosal polypoid lesions with contrast enhancement, indicated by the white arrows

**Fig. 4:**
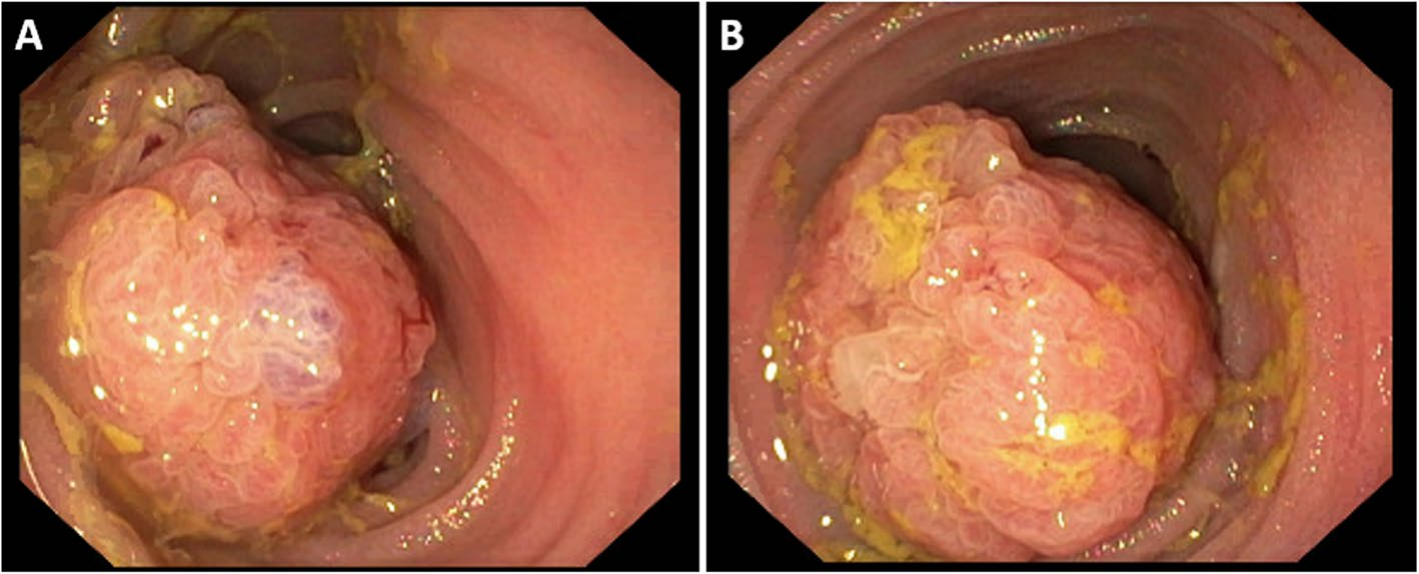
Endoscopic images. Sessile polypoid mass (diameter 5 cm) located in the ileum at 60 cm of the ileostomy entrance (**A**) and impeding passage (**B**)

Interestingly, multiple new intraluminal lesions of the small bowel with contrast enhancement were seen, not corresponding to the intraperitoneal mucinous lesions (Fig. 3). Enteroscopy via end ileostomy revealed a 5-cm sessile polypoid mass at approximately 60 cm with bleeding tendency, impeding further passage and intubation (Fig. 4). With no options for endoscopic resection due to the large size, multiple biopsies were taken and the procedure was terminated.

Histopathological assessment of the polypoid intraluminal lesion revealed intestinal-type neoplastic tissue with hyperchromatic nuclei showing pseudostratification directly adjacent to the mesenchymal stroma. No infiltrative features were present. Additional next-generation sequencing demonstrated an activating mutation for *KRAS* and a mutation for *SMAD4* (Fig. 2d, e). Both mutations were also present in the intraperitoneal localizations of high-grade PMP. No mutations were found in *GNAS*, *BRAF*, *HRAS* and *NRAS*. Based on the clinical, histopathological and molecular findings, the patient was diagnosed with an intraluminal recurrence of PMP located in the ileum.

With no remaining surgical treatment options for the progression of PMP, nor treatment possibilities for anaemia caused by the bleeding intraluminal lesions, the patient was referred back to the initial teaching hospital and received blood transfusions as part of palliative care. During the eighth transfusion, the patient collapsed and sudden cardiac arrest occurred. Cardiopulmonary resuscitation was unsuccessful and the patient deceased shortly hereafter. Post-mortem diagnosis of haemolytic transfusion reaction was excluded by laboratory investigations. The cause of death was determined as a severe gastrointestinal haemorrhage caused by the underlying disease.

## Discussion and conclusions

In this report, we present the case of a unique patient that illustrates the widely varying biologic and clinical behaviour of appendiceal mucinous neoplasms and associated PMP. After undergoing CRS-HIPEC for extensive disease, this patient was diagnosed with high-grade PMP originating from a HAMN. During follow-up, the patient developed intraperitoneal recurrence of PMP together with new intraluminal depositions in the ileum, both lesions with identical *KRAS* and *SMAD4* mutations. Due to progressive PMP, the patient was further treated with palliative intent.

In end-stage disease, peritoneal surfaces of the small bowel can become affected by tumour cells, when excessive amounts of mucinous ascites limit intestinal movements [2]. Appendiceal mucinous tumours are characterized by a ‘pushing’ border growth pattern towards adjacent structures, without infiltration of peritoneum or intestinal organs [21, 22]. Remarkably, this patient developed sessile polypoid masses located in the mucosal wall of the ileum. After extensive histopathological and molecular assessments, the ileal lesions were classified as localization of (recurrent) PMP. There are some reports of PMP originating from colonic or rectal mucinous neoplasms, but none of them describes PMP originating from small bowel mucosa [23, 24].

Despite providing valuable insights into possible dissemination patterns of PMP, a relevant limitation in this report concerns the initial diagnosis of a low-grade mucinous neoplasm. By obtaining only small quantities of mucus with low cellularity, biopsies entail a considerable risk of sampling errors in PMP. In general, the accurate histopathological classification remains uncertain until surgical specimens have been obtained, as in this case.

The pathophysiological mechanism of this unusual site might be explained by the formation of an end ileostomy following CRS-HIPEC procedure. Dissection of the ileum during cytoreduction could induce a risk of tumour cell entrapment within the ileal loop. In time, pseudomyxoma polyps can potentially be formed in the wall of the ileum. However, the end ileostomy was created after cytoreductive surgery and subsequent HIPEC, thereby strongly reducing an exposure risk to circulating tumour cells. In addition, one might expect more similar cases of intraluminal PMP to be present in literature, whereas colostomies and enterostomies are frequently performed procedures after tumour debulking [11, 16, 25].

Another explanation could be the presence of a *SMAD4* mutation. Loss of SMAD4 expression is associated with worse overall survival, and these findings might explain the clinically aggressive behaviour in this patient [26]. *SMAD4* is a protein-coding gene serving as a transcriptional mediator in the transforming growth factor-β (TGF-β) signalling pathway, playing an important role as a tumour suppressor gene [27]. Davidson et al*.* [26] assessed *SMAD4* mutational profiling in 109 patients, and loss of SMAD4 expression was identified in 13 appendiceal mucinous neoplasms, all tumours exhibiting prognostic unfavourable histologic features as high cytological grade, high cellularity and destructive invasion. The intra-abdominal locations of observed destructive invasion were not clarified in this study, impeding further comparison for this patient.

The prognostic relevance of the encountered activating *KRAS* mutation in the patient is not clear. A recent systematic review on mutation status in appendiceal mucinous neoplasms found that *KRAS* mutations are frequently encountered in both low-grade and high-grade primary tumours and their corresponding PMP (76.5% and 74.4% vs 50.4% and 55%, respectively) [28]. While *KRAS* has an important role as a proto-oncogene in the RAS/MAPK signalling pathway by regulating cell proliferation, no significant association was found between *KRAS* mutations and survival outcomes.

Whether the combination of *SMAD4* and *KRAS* mutations increases an invasive capacity remains unknown. Concerning patients with histopathological unfavourable features, high cytological grade and mutational status with unknown potential, the possibility of disease manifestation at unusual sites should always be taken into consideration.

This is the first reported case of PMP with intraluminal recurrence involving the small bowel. This rare disease comprises a wide histopathological and biological spectrum of aggressiveness, as illustrated by the dismal clinical course in our patient. Increased awareness of unusual dissemination patterns may help other clinicians to interpret similar findings, select optimal diagnostic modalities and determine further treatment and prognosis.

## Data Availability

Patient-derived data was extracted from the medical records. Data sharing is not applicable to this article as no datasets were generated or analysed in the present report.

## Abbreviations

PMP: Pseudomyxoma peritonei
AMN: Appendiceal mucinous neoplasm
CRS: Cytoreductive surgery
HIPEC: Hyperthermic intraperitoneal chemotherapy
HAMN: High-grade appendiceal mucinous neoplasm
CT: Computed tomography
